# Mesenchymal Stem Cells and Extracellular Vesicles: Therapeutic Potential in Organ Transplantation

**DOI:** 10.1155/2024/2043550

**Published:** 2024-04-26

**Authors:** Wennuo Pan, Shaohan Li, Kunsheng Li, Pengyu Zhou

**Affiliations:** ^1^Nanfang Hospital, Southern Medical University, Guangzhou 510000, China; ^2^Department of Cardiothoracic Surgery, Nanjing Drum Tower Hospital, Nanjing University Medical School, Nanjing, China; ^3^Department of Cardiovascular Surgery, Nanfang Hospital, Southern Medical University, Guangzhou 510000, China

## Abstract

At present, organ transplantation remains the most appropriate therapy for patients with end-stage organ failure. However, the field of organ transplantation is still facing many challenges, including the shortage of organ donors, graft function damage caused by organ metastasis, and antibody-mediated immune rejection. It is therefore urgently necessary to find new and effective treatment. Stem cell therapy has been regarded as a “regenerative medicine technology.” Mesenchymal stem cells (MSCs), as the most common source of cells for stem cell therapy, play an important role in regulating innate and adaptive immune responses and have been widely used in clinical trials for the treatment of autoimmune and inflammatory diseases. Increasing evidence has shown that MSCs mainly rely on paracrine pathways to exert immunomodulatory functions. In addition, mesenchymal stem cell-derived extracellular vesicles (MSC-EVs) are the main components of paracrine substances of MSCs. Herein, an overview of the application of the function of MSCs and MSC-EVs in organ transplantation will focus on the progress reported in recent experimental and clinical findings and explore their uses for graft preconditioning and recipient immune tolerance regulation. Additionally, the limitations on the use of MSC and MSC-EVs are also discussed, covering the isolation of exosomes and preservation techniques. Finally, the opportunities and challenges for translating MSCs and MSC-EVs into clinical practice of organ transplantation are also evaluated.

## 1. Background

Solid organ transplantation (SOT) is considered a life-saving treatment in patients suffering from terminal organ failures. However, organ shortage limits the applicability of organ transplantation. One way to address this concern is to increase the number of potential donors. Nevertheless, these organs are unlikely to solve the shortage problem completely, because not all donated organs are suitable for transplant, and not all transplant processes will succeed [1]. At present, organ transplantation faces two main problems: (1) graft retrieval, transport, and transplantation inevitably lead to ischemia/reperfusion injury (IRI), resulting in irreversible dysfunction of the graft. The currently accepted standard is static cold storage (SCS), and limitations in treatment manifest clinically as delayed recovery of graft function (DGF) and lead to primary graft dysfunction [2]. Therefore, organs need to be maintained before transplantation and organ preservation technology needs to be improved. (2) The application of new immunosuppressants has effectively controlled cell rejection, but humoral rejection is still an important factor in transplant failure and even the death of recipients. The presence of antibodies in the recipient serum before and after transplant rejection, called donor-specific antibodies, subsequently leads to antibody-mediated rejection. Clinical antibody-mediated rejection prevention and treatment methods mainly use a combination of drugs to remove existing or emerging antibodies, eliminate or inhibit antibody-producing cells, and delay or inhibit antibody-dependent complement damage, but its efficacy needs to be further studied. The diagnosis, prevention, and management of infection are the focus of organ transplantation research, including optimizing immunosuppression, antibody-mediated rejection management, and long-term donor lifespan [3].

Pluripotent mesenchymal stem cells (MSCs) were first identified from bone marrow in 1976 and have now been found in almost all tissues in the human body [4]. MSCs have regenerative, anti-inflammatory, and immunomodulatory properties that are achieved by regulating innate and adaptive immune responses, inhibiting proliferation and function of T, B lymphocytes and natural killer (NK) cells and maturation of dendritic cells, and inducing the production of regulatory T cells. In the future, it will be feasible to establish clinical-grade human MSCs using good manufacturing practices as a universal cell source for clinical immunomodulatory therapy in organ transplantation. At the same time, studies have shown that MSCs have the effect of alleviating graft IRI and inhibiting rejection during the low-perfusion period of rat kidney transplantation. Therefore, the application of MSCs to graft IRI provides new ideas and strategies for organ preservation.

Extracellular vesicles (EVs) are nanoparticles, including exosomes, microvesicles, and apoptotic bodies, that are released into the extracellular microenvironment by eukaryotic and prokaryotic cells and are coated by a phospholipid bilayer membrane. Exosomes contain transmembrane proteins widely distributed from the membrane, which can carry and transport bioactive molecules such as proteins, nucleic acids, and lipids, and also contain many regulatory RNAs in the membrane, thus playing an important role in intercellular material and information transduction. The main functions of exosomes are to inhibit inflammation and apoptosis and promote tissue repair, vascular regeneration, and immune regulation. At present, researchers believe that the application of exosomes in organ transplant immunosuppression can be achieved by designing exosomes that carry targeted ligands and stimulus–responsive factors. Therefore, intravenous infusion of donor-derived exosomes induces donor-specific transplant immune tolerance through the indirect pathway of antigen recognition, which is expected to become an effective means of immunosuppression after organ transplantation. In promoting tissue regeneration, EVs secreted by stem cells can promote cell proliferation by reducing apoptosis and enhancing cell proliferation markers. At the same time, exosomes have unique properties: inherent stability, low immunity, biocompatibility, and good biofilm permeability, enabling them to serve as efficient natural nanocarriers and essential biomarkers in clinical diagnosis [5].

In this review, we will describe the application of MSCs and EVs in organ preservation and coping with IRI. In addition, we will discuss the feasibility of MSCs and EVs in regenerative medicine and immunomodulation. Finally, we will summarize MSCs and EVs-derived biomarkers to evaluate the quality of the graft and predict long-term graft outcomes.

## 2. The Basic Role and Functions of MSCs and MSC-EVs

**Figure 1:**
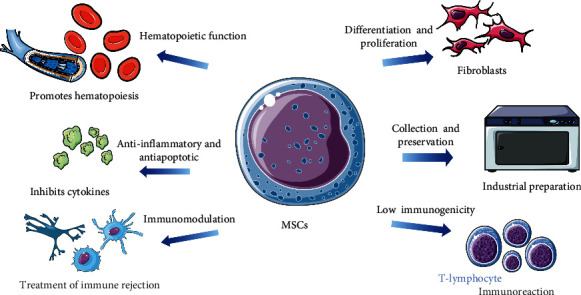
The features and properties of MSC. MSCs have a variety of functions, such as secreting a variety of cytokines, supporting hematopoiesis, strong immune regulation, and promoting the self-repair ability of tissues and organs, which can effectively treat a variety of intractable diseases. The application of mesenchymal stem cells in clinical practice will provide new therapeutic strategies for organ transplantation.

MSCs, the archetypal multipotent progenitor cells which are derived cultures of developed organs, initially discovered from bone marrow in 1976, have been identified in nearly all tissues of the human body now [6]. According to the 2006 consensus of the International Society of Cellular Therapy, the distinctiveness of MSCs relies on the following standard criteria: (1) adherence to plastic surfaces potential to differentiate into astrocytes, (2) regulation and contracts in adipocytes under standard *in vitro* differentiating conditions, (3) surface expression of CiD105, CD73, and CD90, (4) lack of the hematopoietic markers, CD45, CD34, CD14, CD11b, and CD79a, and (5) HLA-DR10. The findings demonstrate that MSCs can suppress graft-versus-host disease and exert profound immunosuppression both *in vivo* and *in vitro* by inhibiting the proliferation and function of a number of immune cell types, including T-lymphocytes, NK cells, dendritic cells (DCs), and promoting the proliferation of regulatory T lymphocytes. Furthermore, with a few expressions of human leukocyte antigen (HLA), MSCs have limited immunogenicity, allowing them to decrease cell immunological function while avoiding allogeneic immune rejection. In this aspect, MSCs are able to secrete anti-inflammatory molecules and inhibit the release of proinflammatory cytokines to attenuate inflammation and promote tissue repair and regeneration *in vivo*, increasing the likelihood of survival of damaged cells [7, 8]. Furthermore, with potent proliferative capacity and multilineage differentiation potential, MSCs can differentiate into cells such as osteoblasts, chondrocytes, adipocytes, myocytes, neuronal cells, hepatocytes, endothelial cells, and stromal cells under appropriate *in vivo* or *in vitro* settings. And through the release of numerous growth factors, MSCs are capable of activating tissue repair with the potential to repair various tissue organs [9, 10] (Figure 1). In recent years, MSCs have been found to release microvesicles that are as biologically active as the cells themselves [11].

MSC-EVs play an active role in cellular microcommunication and the transfer of biological information via internal proteins, microRNAs and lncRNAs [12]. As a type of EVs, exosomes are produced in cells of polyvesicles and are secreted by living cells about the diameter of a living cell. It is a membranous vesicle of 30–150 nm. The separation methods of exosomes mainly include differential centrifugation, density gradient centrifugation, size exclusion chromatography method, filtration method, polymer precipitation method, immune separation, isolation screening method, etc. But for the exosomes on the central nervous system, the mainstream separation method in the literature on bulk research is still the method of ultracentrifugation which accurate and reproducible acquisition of exosomes can be achieved while minimizing protein aggregates and other membrane particles copurification of subs. EVs are biological particles with a heterogeneous phospholipid bilayer that regulate cell communication through molecular cargo delivery and surface signaling. Emerging research indicates that MSC-derived exosomes have the same therapeutic efficacy as MSCs in a variety of diseases while avoiding many of the risks associated with cell transplantation. Their distinct immunomodulatory properties are especially important in graft environments characterized by overactive immune systems [13]. The activated immune response initiates the production and release of inflammatory cytokines, promotes the adhesion and migration of leukocytes during reperfusion, and further increases the inflammatory response. Sustained oxidative damage and inflammatory responses eventually lead to the activation of different cell death programs, further aggravating organ damage. Several studies have found that MSCs can inhibit the inflammatory process during IRI. Cao et al. [14] found that MSCs significantly reduced inflammatory response after donation-after-cardiac-death (DCD) liver transplantation compared to exposure to normothermic machine perfusion (NMP) alone, manifested by reduced levels of IL-1b, IL-6, and TNF-*α*, and reduced expression of molecules associated with the HMGB1 and TLR4/NF-*k*B pathways [14]. Besides, Lonati et al. [15] found that MSC-EVs could induce the expression of various genes involved in anti-inflammatory response and resolution of oxidative stress in rat lungs during NMP. They showed that MSC-EVs could transfer hyaluronan into lung tissue and induce pulmonary production of hyaluronan during NMP [15].

### 2.1. MSCs and MSC-EVs in IRI

The shortage of donors is a major problem in the field of organ transplantation. Today, when there are fewer standard donors, the increased demand for transplantation can only be met by more successful marginal organ transplantation and increased utilization of high-risk organs [16]. The current international standard for organ transplantation is SCS. SCS has the advantages of low cost, simple operation, and ease to use. Low temperature can also prolong the preservation time of organs to a certain extent, but problems such as acid–base imbalance, nutrient deficiency, and accumulation of metabolites after stopping circulation limit the preservation time of organs and affect the quality of donors. More importantly, SCS inevitably causes organ IRI.

IRI occurs during reperfusion of previously surviving ischemic tissue, causing tissue, and cell damage. Disruption of blood supply to organs in the initial stage of ischemia causes an imbalance in metabolic supply and microvascular dysfunction, a decrease in ATP synthesis, a decrease in purine bases, and an increase in intracellular acidic metabolites. When blood supply is restored, sudden perfusion and oxygenation increase organ damage while restoring oxygen supply by activating innate and adaptive immune responses that produce free radicals [17]. To date, IRI remains a clinically unresolved problem and a major cause of graft failure [18], which can lead to DGF, with short- and long-term effects on organ function and survival [19]. Previous studies have shown that transplants from marginal donors are more susceptible to the harmful effects of ischemic injury and hypothermia exposure problems [20]. Therefore, the efficient use of marginal donors urgently requires new preservation technologies.

More recently, machine perfusion has evolved into a new marginal graft preservation strategy. The perfusion solution itself reduces the accumulation of succinate and other metabolites and removes debris and necrotic or apoptotic cells. Furthermore, this technology mimics the physiological state of the organ by continuously perfusing the organ, providing metabolic substrates and oxygen to the donor, removing toxic metabolites in the microcirculation while maintaining circulation, prolonging organ preservation time, and improving graft quality, and reducing the incidence of early graft dysfunction. However, the primary benefit is that oxygenation reduces damage caused by ischemia and anaerobic metabolism [21], and Moers et al. [22] demonstrated that mechanical perfusion was more effective than SCS in protecting dead kidneys from IRI.

**Figure 2:**
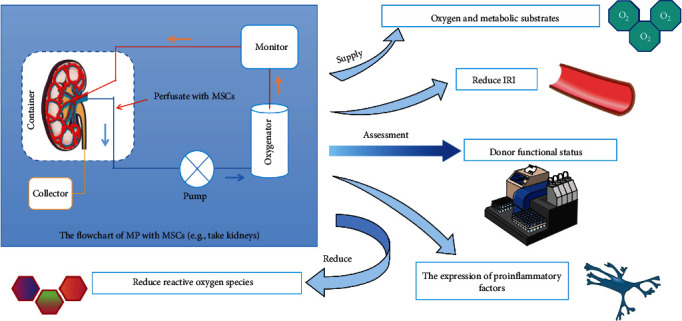
Flowchart of MP with MSC and its advantages. Studies have shown that transplantation of marginal donors is more susceptible to ischemic injury and harmful effects caused by hypothermia. In the context of the extensive utilization of marginal donors, machine perfusion emerged as a new marginal graft preservation strategy.

Recent studies have shown that MSC-mediated has great potential in the treatment and mitigation of IRI in SOT due to its antioxidant, immunomodulatory, and regenerative properties [9]. Simulated experiments have demonstrated that perfusing an *ex vivo* rat kidney with MSCs/EVs can attenuate ischemic injury [23] (Figure 2). The incorporation of bone marrow-derived MSCs into organ preservation fluid has been shown to improve graft dysfunction after heart transplantation [24]. Renal donation after pretreatment of the circulating death of MSC-EVs is associated with a significant reduction in renal ischemic injury. MSCs have also been shown to promote renal recovery from ischemia–reperfusion-induced acute renal failure [25]. Furthermore, Nie et al. [26] found that hu-MSC EVs helped fight hypoxia by protecting pancreatic islet cell masses from hypoxia-induced dysfunction. In animal liver transplantation models, MSCs have been shown to reduce IRI and promote liver regeneration [27].

A growing body of research suggests that machine perfusion combined with MSCs mitigates grafts IRI, especially limbic organs. Machine perfusion provides a unique platform for selective direct injection of these MSCs directly into donor organs, overcoming homing, trafficking, and safety issues [10, 20]. Recent studies by Zeng et al. [28] have shown that normothermic *ex vivo* heart perfusion combined with bone marrow MSCs derived conditioned medium therapy can attenuate myocardial IRI for DCD heart by reducing levels of oxidative stress, inflammatory response, and apoptosis [28]. IRI is often accompanied by local or systemic inflammatory responses called “aseptic inflammation” or “damage-related pattern molecules (DAMPs)” to distinguish them from the inflammatory response to infection. DAMPs activate innate immune response through toll-like receptors (TLRs), especially TLR-4, when the expression and activation of endothelial adhesion molecules, integrins, and selectors increase [19, 29].

## 3. Immunosuppression Therapy

Another highlight of organ transplantation is to improve the longevity and quality of life after organ transplantation and improve the immune tolerance of transplanted organs. The main method is to reduce the body's immunity, but the use of drug inhibitors can have greater side effects because suppressing the body's immunity increases the risk of contracting other diseases. This section reviews the immunomodulatory properties of MSCs in highly complex interactions with immune cells, as well as their ability to inhibit the proliferation and function of a variety of immune cells. At the same time, the application of exosome targeting in organ transplantation therapy, including the method of administration and the amount of injection, was discussed.

### 3.1. MSCs and MSC-EVs in Immunotherapy

Despite the availability of powerful immunosuppressants, acute allograft rejection after organ transplantation is still common. In addition, these immunosuppressants can lead to a significant decrease in the body's immunity, causing complications such as serious infections and malignant tumors. According to earlier research, MSCs control the innate and adaptive immune systems by maturing dendritic cells, suppressing T cells, reducing B-cell activation and proliferation, and blocking the proliferation and cytotoxicity of NK cells [30]. Some of these effects are mediated by soluble factors such as transforming growth factor *β* and prostaglandin E2, among others [31]. Additionally, MSCs hold great promise in the treatment of immune disorders such as host versus graft reaction and allergic disorders, as it is an invaluable cell type for the repair of tissue/organ damage caused by chronic inflammation or autoimmune disorders [32]. In the rat IRI model, injection of EVs secreted from human umbilical cord-derived MSCs reduced the expression levels of the proinflammatory factor interferon (interferon, IFN)-*γ*, tumor necrosis factor (tumor necrosis factor, TNF)-*α*, and interleukin (interleukin, IL)-6, and reduced neutrophil infiltration and respiratory burst. Studies have found that EV secreted by MSCs reduces the proinflammatory factor IL-6 and the CC chemokine ligands (CC chemokine ligand, the surface level of the CCL) 7, enhanced NOD-like receptor protein (NOD-like receptor protein, the expression of the NLRP)12, and NLRP12 is a represof atypical nuclear factor (nuclear factor, NF)-*κ* B signaling pathway of proteins that inhibit the inflammatory response, to effectively address the challenges of DGF and acute rejection (AR). Umbilical cord MSC transfusion has been shown to be feasible for the treatment of acute graft rejection after organ transplantation and may mediate therapeutic immunosuppressive effects [33].

MSCs have powerful immunomodulatory properties in highly complex interactions with immune cells, as well as the ability to inhibit the proliferation and function of a wide range of immune cells. Preinfusion of different regulatory cells, such as MSCs, Tregs, and DCs, is currently an option for transplant recipients [26]. MSCs have been shown to prevent AR in animal livers [22]. Researchers have demonstrated that exosomes have strong immunomodulatory abilities and induce immune tolerance in rat allograft models [34]. Furthermore, Gennai et al. [11] confirmed that MSC-EVs played a beneficial therapeutic role in human lung transplant rejection.

**Figure 3:**
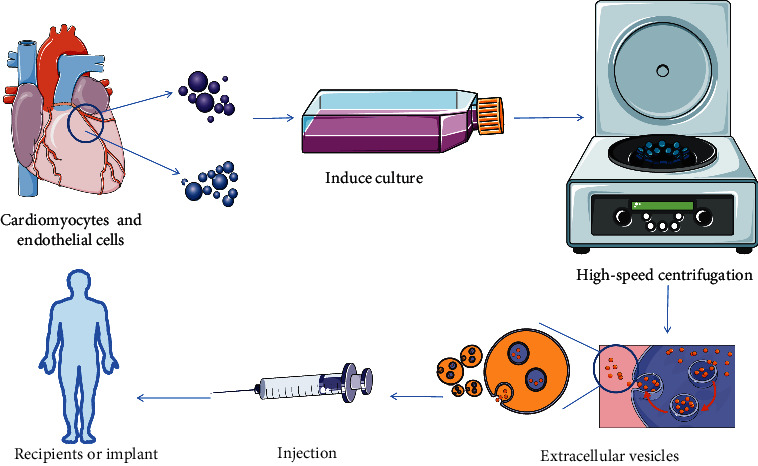
Exosomes extracted from cardiomyocytes are used for immunorejection therapy. Extract cardiomyocytes and endothelial cells from myocardial tissue, perform *in vitro* culture and ultracentrifugation isolation, extract exosomes, and inject them into recipients or graft samples. Exosomes contain T cells that induce inflammation, endothelial activation, AMR, and helper cells, reducing immune rejection of grafts at recipients.

Previous studies demonstrated that MSCs had the immunosuppressive ability to reject after heart transplantation [35]. Casiraghi et al. [36] reported a successful case of a living donor kidney transplant recipient in inducing immune tolerance with autologous MSCs. Morelli et al. [37] confirmed that exosomes function through DCs and elucidated their mechanism of action. Previous studies have shown that DCs in the spleen *in vivo* can target, endo swallow and process blood-derived exosomes, and present allogeneic antigen peptides carried by exosomes [37]. Therefore, intravenous infusion of donor-derived exosomes induces donor-specific transplant immune tolerance through the indirect pathway of antigen recognition, which is expected to become an effective mean of immunosuppression after organ transplantation (Figure 3).

### 3.2. Methods of Administration of MSCs and MSC-EVs in Immunotherapy

MSCs or MSC-EVs are considered to have potential immunomodulatory and repair effects and have been attempted to replace or supplement immunosuppressive agents after organ transplantation. Studies have reported that MSCs therapy after renal transplantation combined with early discontinuation of calcineurin inhibitor (CNI) allows better blood pressure control, reversal of left ventricular hypertrophy, and prevention of progressive diastolic dysfunction without increased risk of graft rejection [38]. In addition, there have been studies to evaluate the efficacy of patients with severe liver failure receiving MSC, treated for ABO-incompatible liver transplantation (ABO-ILT). MSC therapy during the trial was comparable to or even better than rituximab in reducing the incidence of AR. Patients treated with MSCs had significantly lower biliary complication rates and infection rates. The results suggest that MSCs may be introduced as a novel immunosuppressive method for ABO-ILT [39]. In addition, there are studies in 10 liver transplant recipients under standard immunosuppression injected with 1.5–3 × 10^6^/kg third-party unrelated MSCs on postoperative day 3 ± 2 and tried to immunostrip from month 6 to month 12. After MSCs infusion, no patient had impaired organ function (including liver transplantation function). No increased incidence of opportunistic infections or new cancers was detected [40]. However, the use of MSC or EV instead of immunosuppressants is still in the experimental stage, and there is no clear standard treatment regimen or definite treatment cycle. Administration of MSCs or MSCs-EVs is usually an intravenous infusion. Previous studies have shown that the use of MSC or EV contributes to reduce dependence on immunosuppressants [41–45]. But specific treatment options are still evolving and more research is needed to determine best practice. Meanwhile, whether the lifetime use of MSCs like immunosuppressants or MSC-EVs still needs more research and clinical data to determine.

MSCs need to be analyzed by flow cytometry after cell culture. The International Society for Cell Therapy (ISCT) criteria for defining MSCs included CD44, CD29, CD73, HLA-ABC, CD90, CD105 positive, and negative for CD14, CD34, CD45, and HLA-DR. The injection dose of MSCs or MSC-EVs is positively correlated with the subject body weight. The current clinical injection dose is mostly 1.0–3.0 × 10^6^ /kg. The specific dose and number of injections need to be evaluated by the investigator according to the relevant situation and clinical needs. Experimental studies have shown that renal allograft recipients received 2 × 10^6^/kg of human umbilical cord-derived MSCs (UC-MSCs) through the peripheral vein before kidney transplantation, received 5 × 10^6^ cells through the renal artery during the surgical procedure, and the postoperative complications (including DGF and AR) have decreased. This suggests that umbilical cord-derived MSCs can be used as a clinically feasible and safe induction therapy. The appropriate timing and frequency of administration of UC-MSCs may have a significant impact on graft and recipient outcomes [46]. In addition, 10 renal transplant patients received two doses of 1.5 × 10^6^/kg 6 months after transplant and subsequently reduced tacrolimus (level 3 ng/mL) in combination with everolimus and prednisone. Immunosurveillance revealed no biopsy-proven AR or graft loss and renal function remained stable. Studies have shown that giving HLA-selected allograft MSC in combination with low-dose tacrolimus is safe at 6 months after transplantation, at least during the first year after renal transplantation. This lays the foundation for further exploring the efficacy of third-party MSCs in kidney transplantation [47].

## 4. MSCs and EVs in Organ Preservation

One strategy to alleviate the problem of the donor shortage is the utilization of marginal grafts, such as grafts from older donors, or DCD [18]. However, extended criteria donors are especially vulnerable to DGF and AR in organ transplant [48]. To promote the outcomes of marginal donor transplantation, many studies are devoted to finding novel ways to improve graft quality. MSC-mediated therapy is one of the directions, which works through the synergy of EV cargo and soluble molecules. Because of the potential of MSCs and MSC-EVs in organ transplantation, many clinical studies have applied them in the transplantation of marginal donors as an adjunct to drugs (Table 1).

## 5. Restoration of Organ Function and Tissue

One of the major concerned questions in SOT is how to establish long-term allograft survival that is free from immunosuppressive strategies. Regenerative medicine is devoted to replacing and/or repairing tissues and organs for functional restoration, which may represent another promising solution to these critical matters [54]. Numerous researches employing preclinical models of kidney, lung, liver, and heart lesions have demonstrated that MSC-EVs have excellent proregenerative capabilities, mirroring the therapeutic effect of the cells themselves [55–57]. Moreover, MSCs can promote tissue repair and regeneration *in vivo*. Therefore, regenerative medicine may be the next frontier in transplant medicine. The growing evidence in regenerative medicine supports the hypothesis that stem cells exert their therapeutic effect in a paracrine/endocrine manner rather than a direct repopulation of the injured tissues [58–60]. Ischemia causes a rapid depletion of the cellular energy supply because oxidative phosphorylation in mitochondria no longer occurs in the absence of oxygen [61]. Ion transporters are deprived of fuel when there is insufficient ATP, resulting in the intracellular ion accumulation and cell swelling. The use of exosomes naturally produced from a mixture of cardiomyocytes, endothelial cells, and smooth muscle cells enhances cardiac recovery with regenerative benefits equivalent to the injection of human-induced pluripotent stem cells without increasing the risk of complications such as arrhythmias, while also avoiding the risk of tumorigenesis and immune rejection [62] (Figure 3).

Perico et al. [42] discovered that host injection of MSCs before kidney transplantation accelerated the recovery of renal function after surgery. Organ pretreatment also can help to preserve ATP and protect organ energy metabolism during continuous ischemia. Experiments by Jennings et al. [63] demonstrated that pretreatment could alter the energy metabolism of the heart. In addition, transplanting bone marrow-derived cells and cardiac stem cells into failing hearts appeared to provide functional benefits [63]. Pêche et al. [64] have discovered that intravenously delivering donor-type dendritic cell-derived exosomes to heart allograft recipients inhibits acute allograft rejection and results in a considerable extension of allograft survival in a rat model [64]. Compared to the control group, the exosome infusion group showed decreased leukopenia and *γ* interferon (IFN-*γ*) mRNA levels in the graft. *In vitro* experiments confirmed that spleen CD4+ T lymphocyte antidonor T lymphocyte response was reduced [65].

It has been experimentally proven that the administration of microvesicles immediately after renal transplantation could ameliorate IRI in both the acute and chronic stages [66]. Phelps et al. [67] confirmed that bone marrow MSC exosomes can reduce serum creatinine and urea nitrogen levels and significantly improve renal function in the model of acute kidney injury induced by renal ischemia [68]. After renal transplantation, the rats treated with microvesicles derived from human Wharton's Jelly mesenchymal stromal cells improved survival rate and renal function. Li et al. [69] found that urine-derived MSC exosomes could improve the urine microalbumin/creatinine ratio in diabetic rats, reduce mesangial hyperplasia, and reduce apoptosis of tissue cells, thereby improving renal function and delaying the occurrence and development of diabetes [67]. Moreover, an extensive number of publications have highlighted the beneficial effect of EVs in preclinical models of IRI, including promoting renal tubular epithelial cell proliferation and repair in renal IRI models, reducing interstitial inflammatory infiltration, reducing renal fibrosis, and slowing the transition from acute kidney injury to chronic renal failure [69–71].

Immune cell infiltration is an essential step leading to liver injury, which is accompanied by hepatocyte apoptosis, HSC activation, uncontrolled wound-healing pathophysiology, and formation of intrahepatic scar tissue and tumorigenesis [72]. MSCs participate in promoting liver regeneration and repairing liver injury after migrating into injured tissues, undergoing hepatogenic differentiation, reducing apoptosis of hepatocytes, promoting hepatocyte proliferation, and exerting anti-inflammatory and immunoregulatory effects in human and rodent models. Moreover, the transplantation of MSCs and their derivatives effectively promotes liver regeneration to attenuate acetaminophen-induced liver injury [73, 74]. Currently, mesenchymal and induced pluripotent stem cell-derived EVs through engineered cell modification allow targeted therapy to be delivered to organs prior to transplantation, promoting tissue regeneration [75]. In particular, these modification methods offer the potential to explore the effects of several therapeutic strategies such as gene silencing, nanoparticles, and cell therapy in fully functional grafts [76, 77].

## 6. EVs as Biomarkers for Postoperative Rejection

Transplant rejection is a complex immune response against the graft-specific expression of allogeneic antigens recognized as “non-self” by the host immune system. These alloantigen systems are primarily composed of major histocompatibility complex (MHC) molecules and secondary histocompatibility antigens expressed by graft cells. This immune response results in organ rejection [13]. At present, SOT techniques such as kidney, liver, heart, pancreas, and small intestine have become increasingly mature, but rejection and infection are still the two main factors affecting the postoperative survival rate of SOT recipients [78]. The kind of transplanted organs, level of immunosuppression, the requirement for extra antirejection medicine, and occurrence of technical or surgical difficulties are the variables that affect the frequency of infection in SOT patients [14].

There is an urgent need for noninvasive biomarker platforms to monitor immune rejection in organ transplantation. Assessing the quality and risk of grafts and enhancing phenotypic characterization of the pretransplant population can build an early warning mechanism for immunization. Preoperative routine evaluation of receptor age, primary disease, past medical history, graft function level, cardiopulmonary resuscitation, and hypotension and other important indicators affecting organ quality. There are three main types of rejection after organ transplantation: (1) acute cellular rejection (ACR), (2) antibody-mediated rejection, and (3) chronic graft dysfunction.

ACR is the result of adaptive immunity related to MHC mismatch and T cell allogeneic recognition. Cellular immunity plays a leading role in the development of AR [15]. In addition to alleviating the inevitable IRI, the goals of MSCs in SOT include the prevention or treatment of AR [19]. Gunasekaran et al. [79] found that mismatched donor HLA molecules, lung-associated autoantigens, and some miRNA molecules related to injury and inflammation were detected in the secretions isolated from the alveolar lavage fluid and serum of lung transplant recipients who underwent AR, but no corresponding molecular expression was detected in recipient samples with stable function after transplantation [56]. Antibody-mediated rejection is rejection caused by antidonor HLA and/or non-HLA antibodies in the recipient, is another manifestation of AR, can occur alone or in conjunction with ACR, and is one of the main factors leading to chronic rejection and affecting recipient survival. EVs carry a wide variety of immune modulatory molecules, such as cytokines, inhibitory molecules, and growth factors. The packing of nucleic acids and other contents into EVs is coordinated by multiple signals from EVs themselves or the cellular/extracellular environment. As biomarkers are easily detectable in biologic fluids and reflect pathophysiologic conditions, differences in EVs profiles can be considered as a way to predict, detect, and determine the nature and severity of allograft rejection [80]. Exosomes have different RNA profiles in normal and immunocompromised states. Vallabhajosyula et al. [81] showed that exosomal shuttle RNA (esRNA) population characteristics were significantly different between normal and AR patients. The esRNAs profiling mediates metabolic pathways critical to basic lung function, such as collagen biosynthesis, platelet-derived growth factor signaling, cell surface interactions, complement cascade, and nicotinic acetylcholine receptor messaging [34]. The basal pulmonary metabolic pathway is overexpressed in the quiescent state after lung transplantation, while in the AR population, distorted expression profiles of esRNA with antigen treatment and innate and acquired immune activation are shown.

In the xenograft model, long-term follow-up with anti-HLA (human-specific MHC class I) antibodies quantifies the detection of islet-derived exosomes transplanted in recipient blood. Transplanted islet-derived exosomes containing the islet endocrine hormone markers insulin, glucagon, and somatostatin were purified using anti-HLA antibody-conjugated magnetic beads. Immune rejection leads to a marked reduction in exosome signaling from the transplanted islet and different changes in exosome miRNAs and proteome prior to the onset of hyperglycemia [81].

**Figure 4:**
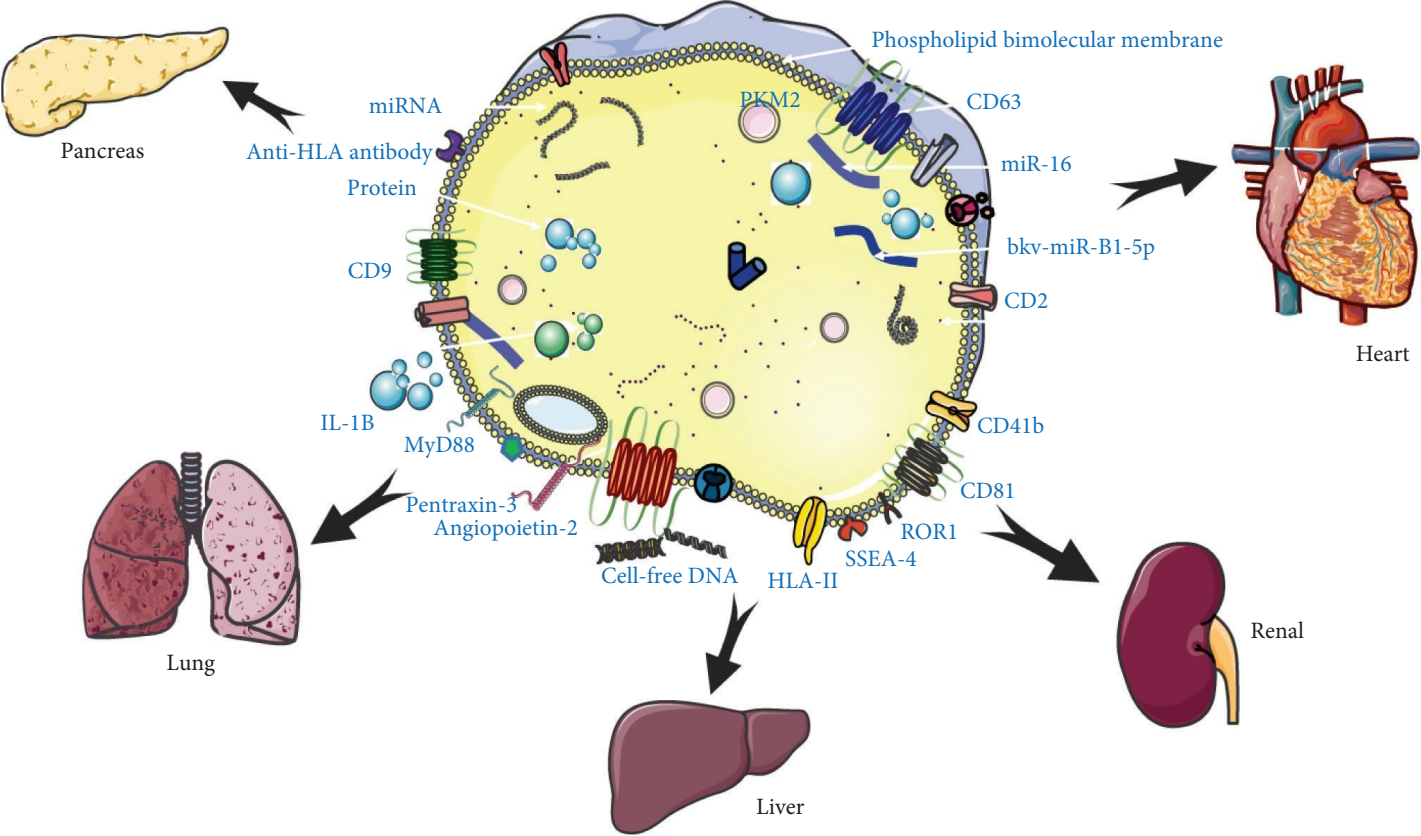
Extracellular vesicles as biomarkers in transplantation. There is accumulating evidence showing that EVs play a pivotal role in the immune system. As they are easily detectable in biologic fluids and contain a specific set of nucleic acids, proteins, and lipids reflecting pathophysiologic conditions, differences in EV profiles can be considered as a way to predict, detect, and determine the nature and severity of allograft rejection. (1) Pancreatic islet: After using an anti-HLA antibody, the islet transplant exosome of the recipients' blood showed significant expression changes in miRNA and the proteome, which enable exosomes to be used to detect rejection before the appearance of hyperglycemia. (2) Heart: Several sensitivity markers (HLA-I, CD2, and SSEA-4 for ACR; ROR1, SSEA-4, HLAII, and CD41b for AMR) can be used for diagnosis and prognosis. Analyzing and combining the different expressions of surface antigens of EV related to the immune system and signal transduction will further improve the diagnostic potential of the inflammatory response, cell survival, and apoptosis. (3) Lung: The expression of molecules such as donor-derived cell-free DNA, cytokines, HIF-1*α*, and MyD88 in exosomes are isolated from the not stable lung transplant recipients with BOS, which can be predicted and taken adequate measures in advance. Also, the cell-free DNA, long pentraxin-3, angiopoietin-2, and IL-1B are other biomarkers to be used to diagnose and treat inflammatory responses. (4) Renal: bkv-miR-B1-5p and bkv-miR-B1-5p/miR-16 in exosomes are two miRNAs encoded by PVAN that serve as biomarkers of early and late renal transplant dysfunction, and they both exhibit very high discrimination ability for kidney transplant complications.

Serum creatinine, particularly in patients with DGF, is widely recognized as an ineffective and advanced marker for predicting graft recovery after kidney transplantation [82]. However, only when the filtration rate during the effective period is reduced by more than 50%, the blood creatinine concentration will increase significantly. Therefore, there is an urgent clinical need for indicators that can indicate an early decline in renal function. Studies have found that fetal globulin A changes significantly in the early stage of (2–8 hr) renal IRI rat injury [83]. Further clinical studies have shown that fetal globulin is also elevated in the urinary exosomes in kidney transplant recipients. In the early postoperative period after kidney transplantation, serum and urine neutrophil gelatinase-associated lipid carry proteins predict graft function delay and graft long-term survival [84]. Currently, the diagnosis of AR after transplantation and drug toxicity of CNIs relies on the renal puncture, and clinically hope to find new diagnostic methods to reduce invasive procedures in patients. A variety of water transporters and ion transporters, such as aquaporin-2 and Na^+^-Cl^−^ cotransporters, are found in urine exosomes, and their abundance can later reflect the transport activity of the renal tubules, so they can be used as markers to evaluate renal tubular function. Other subsequent causes include transplanted glomerulopathy, recurrent glomerulonephritis, and renal artery stenosis [85]. Therefore, judging the immune status and function of organs also needs to combine a variety of factors other than exogenous inclusions. Allogeneic graft dysfunction, whether in the early or late posttransplant period, requires prompt evaluation to determine its etiology and subsequent management. AR, drug toxicity with CNIs, and BK virus nephropathy can occur early or late. Other common recurrent conditions after kidney transplantation include transplanted glomerulopathy, recurrent glomerulonephritis, and renal artery stenosis, which are not only manifested in exosome-specific changes but are also involved in the expression of other bioactive molecules [85]. Therefore, judging the immune status and function of organs also needs to combine a variety of factors other than exogenous inclusions (Figure 4).

## 7. Evaluation and Limitations of Clinical Translation of MSCs and EVs

### 7.1. Research and Clinical Application of MSCs in SOT

In the transplantation treatment of tissues and organs, MSCs mainly play three roles: inhibiting immune rejection and inducing immune tolerance of allogeneic transplanted organs, promoting functional repair of tissues and organs and preventing deterioration, and reducing the total dose of induced or maintained immunosuppression. At present, most of the applications of MSCs in the field of tissue and organ transplantation are in the phase I clinical stage, although there are inherent design limitations in this stage of research (including sample size and treatment regimens), MSCs have shown promising efficacy in protecting grafts from chronic rejection and promoting immune tolerance, and it is worth continuing to carry out large-scale clinical studies.

In 2011, a phase I clinical study was conducted by the team of Prof. Giuseppe Remuzzi in Italy to evaluate the safety and feasibility of MSCs in two living donor kidney transplant recipients [86]. In this clinical study, two kidney transplant patients were transfused with autologous bone marrow MSCs at a dose of (1–2) × 10^6^/kg 7 days after transplantation, and immune monitoring showed a gradual increase in Treg fraction, a significant decrease in the percentage of circulating CD8+ memory T cells, and a decrease in donor-specific T cell alloreactivity. Long-term follow-up highlighted a sustained increase in the ratio between Treg and CD8+ effector T cells in one of the patients, who gradually reduced immunosuppression and achieved discontinuation of immunosuppression for more than 2 years, demonstrating that a single dose of MSCs can induce long-term immune tolerance. Four patients were given back autologous bone marrow MSCs before kidney transplantation and 30 days after transplantation, and the MSCs also exerted good antirejection effect while increasing the number of Treg cells. Subsequently, the clinical study of MSCs in the treatment of two kidney transplant patients was continued, and the reinfusion of MSCs was given before kidney transplantation, which also achieved good results, i.e., reducing rejection and increasing the number of Treg cells.

Similar to kidney transplantation, MSCs are also used in liver transplant patients to induce surgical tolerance, suppress AR, and treat ischemic biliary tract lesions. A recent controlled clinical study by Zhang et al. [87] found that a single infusion of 1 × 10^6^/kg umbilical cord MSCs (three injections in one patient) in addition to conventional immunosuppressants was used to treat acute liver transplant rejection [87]. At the end of 12 weeks of follow-up, patients in the MSCs-treated group showed lower hepatic aminotransferase levels and histological improvement compared to the control group, especially an increase in the number of circulating Treg cells in the body and an increase in the proportion of Treg/Th17 at 4 weeks. After liver transplantation, ischemic biliary lesions appeared, and 12 patients received six intravenous infusions of umbilical cord MSC (1.0 × 10^6^/kg, weeks 1, 2, 4, 8, 12, and 16); total bilirubin, *γ*-glutamyl transferase, and alkaline phosphatase all decreased compared to baseline; 64.3% (45/70) of the patients in the control group received interventional therapy, while only 33.3% (4/12) of the patients in the MSCs treatment group received interventional therapy, which significantly reduced the need for interventional therapy and improved the survival rate of transplanted liver.

In lung transplant recipients, if chronic lung dysfunction occurs after the transplant, this will severely affect the survival of the transplanted lung. A team at the University of Queensland, Australia, led a phase I trial evaluating the safety and feasibility of four allogeneic bone marrow MSCs (2 × 10^6^/kg twice a week for 2 weeks) infusion in 10 patients with progressive chronic lung transplant dysfunction [88]. MSCs treatment was well-tolerated with no adverse events involving hemodynamic or gas exchange, two patients died from failure to correct pulmonary dysfunction, and the rest showed improvement in pulmonary function.

In fact, there is an optimal dosage for the efficacy of MSC therapy, and the infusion dose should be given based on the severity of each patient's disease, their own condition, and injection method. At present, clinical trials often use a single infusion dose of (1–2) × 10^6^/kg MSC, with a 4-week interval for reinjection. Three to six injections are used as a course of treatment (some experiments use single injections). In addition, attention needs to be paid to the source of cells, cell survival rate, and cell growth activity, which are key standards for high-quality stem cells. In addition, the therapeutic application of autologous bone marrow MSCs is not advocated, as donor age and pathological status can greatly affect the cell quality and quality effect of MSCs.

### 7.2. Research and Clinical Application of MSC-EVs in SOT

Some studies have found that MSCs may not function in a cellular manner, but rather through EVs [89]. In addition, EVs are smaller than cells and can penetrate the blood–brain barrier. Therefore, MSC-EVs may have advantages over their parent MSCs in inducing immune regulation, anti-inflammatory, and tissue regeneration effects during systemic administration [90]. At present, EVs treatment is a promising method for inhibiting allograft rejection and improving transplant outcomes. In terms of source, exosomes have good stability and are easy to obtain. It also plays a role in prolonging graft survival time and reducing immunosuppressive load, providing direction for future cell-free therapy.

An experimental study was conducted to deliver MSC EVs to a rat DCD kidney model during low-temperature mechanical perfusion and evaluate the extent of renal ischemic injury. This experiment isolated EVs from 3 million MSCs and added the separated EVs to the infusion solution. The results showed that compared to Belzer perfused kidneys, the overall renal injury score of MSC-EV perfused kidneys was significantly lower [23]. In animal experiments, EVs are usually isolated from (3–20) × 10^6^ MSCs. A cell secretes approximately 2,000 EVs and typically injects (1–1.5) × 10^9^ EVs into the animal body at once [91].

A MSC cell secretes approximately 2,000 EVs, and the specific number of EVs extracted in the laboratory is also influenced by factors such as cultivation methods and extraction methods. A study has prepared EV products derived from single donor bone marrow MSCs as investigational product (IP) based on FDA approved treatment regimens [92]. Inject 15 mL of IP intravenously to transplant patients on days 0, 2, and 4, repeating four times for each patient. There were no adverse events related to IP as a result. All patients showed improvement in clinical symptoms within 24 hr of administration of the investigational drug and complete histological resolution of graft inflammation/rejection within 7 days of administration of the investigational drug. Therefore, the systematic use of EVs derived from MSCs can achieve histological clearance of graft inflammation.

However, there are still many unknowns about the mechanism by which MSC EVs play a communication role between cells, especially in the field of organ transplantation, where the specific patterns and interrelationships between donor organs and recipient bodies are not yet clear. Therefore, before applying MSC EVs in clinical practice, more in vivo studies are needed to elucidate the optimal cell source, dosage, and treatment plan.

### 7.3. Prospects and Limitations of Clinical Application

MSCs and MSC-EVs offer new strategies for organ transplantation in organ preservation, immune rejection therapy, targeted biological drug delivery, and biomarker detection, but there are still challenges in translating them into organ transplant clinical practice. Among them, developing strategies to limit the progression of IRI is the basis for successful transplantation. Combining bioactive molecules such as MSCs with machine perfusion has the potential to significantly reduce tissue and organ damage. However, the special law and interrelationship between donor organs and recipients are not clear, and the damage repair effect and regeneration mechanism of MSCs have not been fully elucidated, so it is necessary to continuously improve the application scope and standards of mechanical perfusion technology to promote the intervention and repair of donors and optimize marginal donors. In addition, immune rejection has always been the main cause of transplant failure in organ transplantation, and the prevention and treatment of rejection mainly include tissue matching, immunosuppression, and immune monitoring. Although many studies have revealed the potential immunosuppressive effects of MSCs and MSC-EVs in transplanted diseases by regulating macrophages and T cells; however, the immune response and immunomodulation of organs are a comprehensive process of various innate and adaptive immune responses. Therefore, it is necessary to explore whether MSC-EVs affect other immune cells, such as DCs, NK cells, and B cells, and how they regulate cell-to-cell communication between various immune cells. In the detection and diagnosis of immunomodulation, exosomes have higher specificity and sensitivity as biomarkers due to their good stability and easy availability. However, at present, in the field of organ transplantation, the properties, inclusions, and distribution of exosomes through a complex *in vivo* and *in vitro* environment still need to be elucidated. Therefore, the clinical application of MSCs and MSC-EVs should pay attention to three points: first, the specification of cell culture conditions; the second is the optimization of exosome isolation and purification methods; and the third is the identification and follow-up detection in the immunomodulation of organ transplantation.

## 8. Conclusions

Improving organ availability and reducing immune rejection are major challenges in the field of organ transplantation. Among recent innovative strategies, the clinical application of MSCs and EVs has great potential. The application of EVs in transplant perfusion solutions can improve the vitality and function of donor organs, reduce organ IRI, and accelerate postoperative tissue repair. At the same time, MSCs regulate the rejection after transplantation through direct, indirect, or semidirect allogeneic recognition pathways, and induce donor-specific transplant immune tolerance, which is expected to become an effective means of immunosuppression after organ transplantation. In addition, exosomes with specific recognition properties can be used as biomarkers and also have beneficial effects on targeted drug delivery. Consequently, MSCs, EVs, and exosomes will have a wide range of applications in future organ transplantation and are ideal candidates for different aspects of the transplant process.

## Figures and Tables

**Table 1:** Recent summary of the clinical practice of MSCs and MSC-EVs in organ transplantation.

Source	Occasion	Type	Result	References
Autologous BM-MSC	Postoperative	Hematopoietic stem cell transplantation	It has been determined that administering autologous MSCs four times a week is safe for the treatment of refractory GVHD after malignant hematological disease HCT	[49]

Autologous BM-MSC	Postoperative	Renal transplant	Peripheral blood immune cells in kidney transfer recipients after mesenchymal stem cell therapy and tacrolimus discontinuity may help improve therapeutic strategies using mesenchymal stem cells to reduce the use of calcium inhibitors	[45]

Autologous BM-MSC	Postoperative	Renal transplant	Plays an immunosuppressive role	[43]

Autologous BM-MSC	Postoperative	Renal transplant	Prompt for systemic immune suppression	[50]

Autologous BM-MSC	Postoperative	Renal transplant	Prove that MSC treatment combined with early cessation of CNI is associated with better blood pressure control, reversal of left ventricular hypertrophy, and prevention of progressive diastolic dysfunction after kidney transplantation	[38]

Autologous BM-MSC	Preoperative	Renal transplant	Pretransplant infusion of MSC has a safer advantage than posttransplant (day 7) cell administration regimen	[42]

Autologous BM-MSC	Preoperative and postoperative	Renal transplant	Autologous MSC infusion is safe and feasible in patients undergoing living kidney transplantation	[51]

Allosome UC-MSC	Postoperative	Liver transplantation	Effective in preventing antibody-mediated humoral rejection	[39]

Allosome BM-MSC	Postoperative	Liver transplantation	MSC infusion on day 3 after liver transplantation had no significant side effects	[40]

Allosome BM-MSC	Postoperative	Renal transplant	The safety of the infusion 6 months after transplantation of HLA-selected allogenic MSCs	[47]

Allosome BM-MSC	Preoperative	Liver transplantation	Preoperative infusion of MSC in liver transplant recipients is safe and causes mild positive changes in immunomodulatory T cells and NK cells in peripheral blood	[52]

Allosome BM-MSC	Preoperative	Renal transplant	Elucidate UC-MSCs in allogeney clinical value of preventing both DGF and acute rejection in organ kidney transplantation	[53]

Allosome BM-MSC	Preoperative	Renal transplant	Attenuate graft loss	[46]

In organ transplantation, the type of mesenchymal stem cells and MSC-EVs and the timing of treatment are critical. The table provides a summary of the methods currently used in clinical trials and animal studies.

## Data Availability

Previously reported (statistics) data were used to support this study. These prior studies (and datasets) are cited at relevant places within the text as references [1–92].

## Abbreviations

MSCs: Mesenchymal stem cells
EVs: Extracellular vesicles
MSC-EVs: Mesenchymal stem cells-derived extracellular vesicles
SOT: Solid organ transplantation
DCD: Donation-after-cardiac-death
DGF: Delayed graft function
NK: Natural killer
DCs: Dendritic cells
SCS: Static cold storage
AR: Acute immune rejection
IRI: Ischemia/reperfusion injury
MHC: Major histocompatibility complex
ACR: Acute cellular rejection
NMP: Normothermic machine perfusion
HLA: Human leukocyte antigen
esRNA: Exosomal shuttle RNA
ABO-ILT: ABO-incompatible liver transplantation
DCD: Donation-after-cardiac-death
CNI: Calcineurin inhibitor.
